# Anti-Inflammatory Effect of a TCM Formula Li-Ru-Kang in Rats With Hyperplasia of Mammary Gland and the Underlying Biological Mechanisms

**DOI:** 10.3389/fphar.2018.01318

**Published:** 2018-11-20

**Authors:** Yingying Wang, Shizhang Wei, Tian Gao, Yuxue Yang, Xiaohua Lu, Xuelin Zhou, Haotian Li, Tao Wang, Liqi Qian, Yanling Zhao, Wenjun Zou

**Affiliations:** ^1^College of Pharmacy, Chengdu University of Traditional Chinese Medicine, Chengdu, China; ^2^Department of Pharmacy, 302 Military Hospital of China, Beijing, China; ^3^Affiliated Hospital of Chengdu University of Traditional Chinese Medicine, Chengdu, China; ^4^Department of Traditional Chinese Medicine, First Affiliated Hospital of Chinese People’s Liberation Army General Hospital, Beijing, China

**Keywords:** Li-Ru-Kang (LRK), hyperplasia of mammary gland (HMG), inflammatory responses, oxidative stress, MAPK

## Abstract

Li-Ru-Kang (LRK), a formula of eight traditional Chinese medicines (TCM), has been used to treat hyperplasia of mammary gland (HMG) in TCM clinics. However, how LRK works in HMG patients is unclear. To explore the possible mechanisms of LRK against HMG, the network pharmacology was used to screen the potential targets and possible pathways that involved in LRK treated HMG. Rat HMG model induced by estrogen and progesterone was used to further verify the effects of the key molecules of LRK selected from the enriched pathways on HMG. Nipple heights and diameters were measured and uterus index was calculated. The histopathological changes of mammary gland tissue were detected by hematoxylin-eosin (H&E) staining. Western blot was used to detect the phosphorylation of ERK, JNK, and P38. And immunohistochemistry staining was performed to evaluate the levels of estrogen receptor α (ERα), progesterone receptor (PR), nuclear factor-(NF-)κB (p65), interleukin-1β (IL-1β), tumor necrosis factor α (TNF-α), cyclooxygenases 2 (COX-2), inducible nitric oxide synthase (iNOS), 8-hydroxy-2′deoxyguanosine (8-OHdG), and nitrotyrosine (NT). Our results indicate that LRK treatment rescues significantly nipples height and diameter, decreases uterus index and ameliorates HMG. LRK treatment also markedly attenuates the over-expression of IL-1β, TNF-α, COX-2, and iNOS, and suppressed the formation of 8-OHdG and NT. Furthermore, LRK treatment significantly inhibits the phosphorylation of JNK, ERK, and p38 and expression of NF-κB (p65), interestingly, LRK treatment has no effect on the expression of ERα and PR. Our data suggest that the LRK treatment protects the mammary glands from the damage of oxidative stress and inflammation induced by estrogen and progesterone, via suppresses of MAPK/NF-κB signaling pathways without affecting on the expression of ERα and PR.

## Introduction

Hyperplasia of mammary gland (HMG), a common disease, occurs among middle-aged women with high frequency (Jia et al., 2017). It is a kind of pathological hyperplasia of lobules of mammary gland induced by the disorder of estrogen and progesterone (Bennett et al., 1990; Zhang et al., 2016). The morbidity of HMG is increasing nowadays with a risk of breast cancer (Chen et al., 2015). Tamoxifen is an estrogen antagonist with antiproliferative effects. It has been widely used for breast cancer as well as HMG (Cline et al., 1998; Li et al., 2018). However, some women do not tolerate tamoxifen and have a risk of side effects (Kleinberg et al., 2011; Martinez de Dueñas et al., 2014). Therefore, it is important to discovery safer and more effective drugs with minimum side effects for HMG.

Traditional Chinese medicine (TCM) has been frequently used as alternative treatments for various types of diseases. For complicated or multi-factorial diseases, emerging evidence indicates that multiple drugs that have common or different pharmacological targets often display better therapeutic efficacy than a single medication (Roth et al., 2002). The formulae with multiple herbs exert therapeutic efficacies through the synergistic effects of their multiple ingredients via multiple targets (Che et al., 2013; Shao and Zhang, 2013).

Li-Ru-Kang (LRK) consisted of Ostreae Concha (Ostrea gigas Thunberg.), Cervi Cornu [Alpinia aquatica (Retz.) Roscoe.], Polygoni Multiflori Radix [Polygoni Multiflori (Thunb.) Moldenke.], Curcumae Radix (Curcuma aromatica Salisb.), Cremastrae Pseudobulbus Pleiones Pseudobulbus [Cremastra appendiculate (D. Don) Makino.], Bupleuri Radix (Bupleurum chinense DC.), Prunellae Spica (Prunellae vulgaris L.) and Glycyrrhizae Radix et rhizoma (Glycyrrhizae uralensis Fish. ex DC). LRK is a Chinese preparation formulated through TCM principles of tonifying the kidney, smoothing the liver, dissipating nodule and activating blood circulation. LRK has an overall regulatory effect on HMG. The randomized trials of LRK showed that LRK had favorable effect in the treatment of HMG (Qian et al., 2007; Li et al., 2013). Studies showed that LRK could improve the histological lesions in mammary gland, uterus and ovaries (Qian et al., 2005). LRK also affects abnormal secretion of sex hormones in model of HMG induced by estradiol benzoate. The serum levels of estradiol (E2) and prolactin (PRL) were decreased and progesterone (P) was increased remarkably by LRK (Qian et al., 2004). Our previous study explained the modulatory properties of LRK treatment on HMG using metabolomics and network pharmacology analyses, showed the therapeutic effects of LRK on HMG (Wei et al., 2018). However, the biological mechanisms of LRK for HMG are still blur.

Network pharmacology, a technology for system biology study, could clarify the potential mechanisms of complicated ingredients through large data set analysis. In that TCM formula has been thought to be multi-ingredients and multi-targets, network pharmacology is a suitable approach to meeting this challenge and determining the mechanism on LRK for treating HMG (Che et al., 2013; Tao et al., 2013; Mao et al., 2017).

Therefore, in the present study, network pharmacology approach combined with molecular biology was performed to further investigate the active ingredients and the underlying mechanism of LRK for the treatment of HMG.

## Materials and Methods

### Database Construction

The chemical structures of the compounds in LRK were obtained from TCM Database@Taiwan (TDT)^1^ (Chen, 2011) and Traditional Chinese Medicine Systems Pharmacology (TCMSP) database^2^. All compounds were selected according to selection criteria [oral bioavailability (OB) ≥ 30 and drug-likeness (DL) ≥ 0.18], as suggested by TCMSP (Liu et al., 2013). Known compound targets were collected from Herbal Ingredients’ Targets Database (HIT)^3^ (Ye et al., 2011), and the putative targets from these were screened out from Therapeutic Targets Database (TTD)^4^ through structural similarity comparison (Chen et al., 2002). Gene and protein targets associated with HMG therapy were collected from the Online Mendelian Inheritance in Man (OMIM)^5^ database. Other interaction proteins of the aforementioned targets were obtained from Database of Interacting Proteins (DIP)^6^, and different ID types of the proteins were converted to UniProt IDs.

### Network Construction and Analysis

To provide the scientific and reasonable interpretation of the complex relationships between the compounds and targets associated with HMG, network analysis was carried out. The compound-target-disease network was constructed using candidate compounds, potential targets and HMG significant targets. The network was performed using Cytoscape 3.5.1 software (National Institute of General Medical Sciences, United States). The topological features of each node in the network were calculated by “Degree”, “Betweenness centrality”, and “Closeness centrality” (“Degree” values twofold greater than the median value of all the network nodes, “Betweenness centrality” and “Closeness centrality” value greater than the median value of all the network nodes) (Wang J.B. et al., 2018). Targets with higher value were screened as the candidates for HMG.

### Plant Material

LRK consisted of *Ostreae Concha* (*Ostrea gigas* Thunberg.), *Cervi Cornu* [*Alpinia aquatica* (Retz.) Roscoe.], *Polygoni Multiflori Radix* [*Polygoni Multiflori* (Thunb.) Moldenke.], *Curcumae Radix (Curcuma aromatica* Salisb.), *Cremastrae Pseudobulbus Pleiones Pseudobulbus* [*Cremastra appendiculate* (D. Don) Makino.], *Bupleuri Radix (Bupleurum chinense* DC.), *Prunellae Spica (Prunellae vulgaris* L.) and *Glycyrrhizae Radix et rhizoma (Glycyrrhizae uralensis* Fish. ex DC) which were purchased from Heyanling, Co., Ltd. (Beijing, China). The origin and quality of the 8 herbs were identified according to the Chinese Pharmacopeia (2015 Edition). The eight TCMs of LRK were composited with the weight ratio of 30: 12: 12: 10: 10: 9: 9: 6. At first, *Cervi Cornu* slice were smashed into powder. Then it was decocted with appropriate amount of water and kept slightly boiling for 1 h. Next, the filtrate of *Cervi Cornu* was obtained and combined with the rest of TCM proportionally, followed by another 1 h heat extraction with boiled water (1/10, weight/volume) three times. The final yield of powder to raw materials was about 9.2%.

### Reagents

The antibodies of phosphorylation-MAPK Family Antibody Sampler Kit used for western blot were purchased from Cell Signaling (United States) (#9910). And nuclear factor- (NF-)κB (p65) (XCJ36131), cyclooxygenases 2 (COX2) (06416080202), inducible nitric oxide synthase (iNOS) (16716110102), interleukin-1β (IL-1β) (01016051201), tumor necrosis factor α (TNF-α) (60291-1-Ig), estrogen receptor α (ERα) (AF6058), and progesterone receptor (PR) (37917011102) for immunohistochemistry were purchased from Wu han goodbio technology Co., Ltd (China). Antibodies of 8-hydroxy-2′deoxyguanosine (8-OHdG) (GR3173165-3), nitrotyrosine (NT) (GR174728-22), GAPDH (AC001) were purchased from Abcam (the United States).

### Animals and Administration

Female SD rats weighting 180–220 g [license number: SCXK-(A) 2012–0004] were obtained from the laboratory animal center of the Military Medical Science Academy of the People’s Liberation Army (PLA). They were maintained separately at animal experimental center of 302 Hospital of People’s Liberation Army (Beijing) with a specific pathogen free (SPF) environment (24°C, 65% humidity, 12 h day/night). The rats were randomly divided into six groups. All rats except for control group were intramuscularly injected with estrogen at a dose of 0.5 mg/kg/d for consecutive 25 days. All rats but control group were intramuscularly injected with progesterone at a dose of 5 mg/kg/d in the following 5 days (Wang et al., 2011). LRK (0.056, 0.112, 0.224 g/kg/d for the low, medium and high dose, respectively) and tamoxifen (Yangtze River Pharmaceutical Co., Ltd. 5 mg/kg/d) were dissolved in normal saline and intragastrically administered to rats, respectively, except for control and model group for 30 days. The rats were sacrificed 12 h after the last administration. All animal studies have been approved by the Ethical Committee of 302 Military hospital of China. The blood, mammary gland and uterus were collected. The blood was centrifuged at 3500 rpm for 15 min to separate the serum without hemolysis. Serum and the rest of mammary gland tissue for hematoxylin-eosin staining were stored at -80°C.

### Measurement of Body Weight, Nipple Height and Diameter, and Uterus Indexes

Body weight, nipple height and diameter of all rats were recorded after administration. Uterus index (Wang et al., 2011) was calculated by the following formulae:

$$\text{Uterus index}={\text{W}}_{\text{uterus}}(\text{mg})/{\text{W}}_{\text{body}}(\text{g})$$

W_uterus_ and W_body_ stand for the average weight of uterus and body weight of rats.

### Histopathological Evaluations and Immunohistochemical Observation

The mammary gland tissues obtained from the experimental rats were fixed in the 10% neutral buffered formalin and then embedded in paraffin. Subsequently, the embedded mammary gland tissues were cut into thin slices and disposed using hematoxylin-eosin (H&E) staining.

Immunohistochemical analysis was performed using deparaffinized mammary sections. The sections were immersed in freshly prepared 2% H_2_O_2_ at room temperature for 25 min and blocked with 5% rabbit serum for 30 min. Then the primary antibody [NF-κB (p65) (1:100), -COX-2 (1:1000), -TNF-α (1:200), -IL-1β (1:400), -iNOS (1:1000), -NT (1:100), -8-OHdG (1:100), -ERα (1:200), or -PR (1:200)] was added and incubated at 4°C overnight. After being washed with PBS, the sections were treated with the secondary antibody conjugated with horseradish peroxidase at room temperature for 50 min. Then, they were immersed in diaminobenzidine (DAB) for 3 min. The hematoxylin-stained sections were dehydrated by ethanol. Stained areas of the sections were visualized using an optical microscope at ×200. Image analysis software Image-Pro Plus 6.0 was used to select the yellow area of the immunohistochemical reactant on the image and then the mean integrated optical density (IOD) of these areas was measured.

### Western Blot Analysis

The mammary expressions of p-P38 p-JNK and p-ERK were evaluated by western blot analysis. Mammary gland tissue of rats was homogenized and subsequently lysed by tissuelyser (Shanghai Jingxin Industrial Development Co., Ltd, Shanghai, China) with RIPA buffer containing a protease inhibitor mixture. The protein was distilled and then centrifugated at 12,000 rpm and 4°C for 10 min to separate debris. Protein concentration was determined using the BCA protein assay kit. Protein samples (25 μg) were separated by SDS-polyacrylamidegel electrophoresis and transferred to a PVDF membrane by electrophoretic transfer. Transferred membranes were blocked for 1 h at room temperature with 2% BSA in Tris-buffered saline containing 0.1% Tween 20 (TBST), and then incubated overnight at 4°C with different primary antibodies [anti-p-P38 (1:1000), p-JNK (1:1000) and p-ERK (1:1000)]. After washes with TBST 4 times, the membranes were incubated with horseradish peroxidase-conjugated secondary antibody (1:3000) in TBST with 2.5% nonfat milk for 1 h at room temperature. Western blots were developed on films using the enhanced chemiluminescence technique. Quantification of bands was determined by densitometric analysis using Bio-Rad Quantity One. The data were normalized using GAPDH (1:3000) as an internal control.

### Statistical Analysis

Data were presented as means ± SD. and were analyzed using the IBM SPSS Statistics 21. Data among groups were analyzed with ANOVA. *P* < 0.05 and *P* < 0.01 were considered statistically significant.

## Results

### Prediction for the Direct Targets of LRK on HMG

**FIGURE 1:**
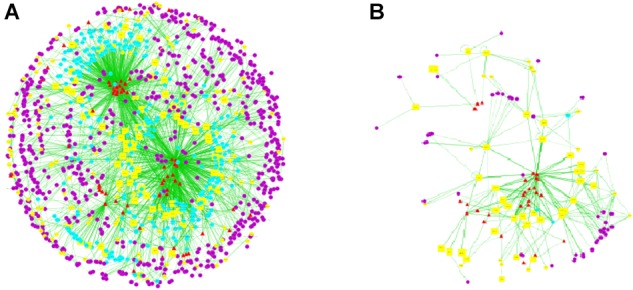
Compound-target-disease network of LRK. **(A)** The main network of “compound-target-disease” of LRK. **(B)** The sub-network with direct targets and the corresponding ingredients. Notes: The red triangles represent active chemical constituents of LRK, the blue dots represent the indirect targets for drugs, the yellow dots represent the targets of the specific disease of HMG, the yellow squares represent the common targets of herbs and HMG, and the purple dots represent the interactional proteins with the targets of HMG and drugs.

A total of 72 chemical constituents of LRK, 351 drug targets, 213 HMG targets and 358 interacting proteins were obtained from TDT, HIT, TTD, OMIM, and DIP. Then, the ingredients, drug targets, disease targets and interacting proteins were connected for interaction network of “compound-target-disease” construction, which was presented with color-coded nodes (Figure 1A). In the network, the yellow squares represented the direct targets which were the common targets of LRK and HMG. The common targets were the key targets for LRK on treating HMG as well as the relatively important targets screened for further research.

Subsequently, the topological parameters values of each target in the network including “Degree”, “Betweenness centrality”, and “Closeness centrality” were analyzed for the important key protein targets and related signaling pathways screening. The results showed that 13 direct targets were selected according to their Degree twice greater than the median (>14), Betweenness Centrality and Closeness Centrality greater than the median (Betweenness Centrality value >0.004; Closeness Centrality value >0.28) (Table 1). Additionally, COX2 and NF-κB might be the most important targets of LRK on treating HMG due to their highest degree.

**Table 1:** Topological parameters of Li-Ru-Kang (LRK) on HMG obtained from network pharmacology analysis.

UniProt ID	Direct target	Degree	Betweenness centrality	Closeness centrality
P35354	PTGS2 COX2	34	0.020	0.302
Q04206	RELA NFKB	28	0.026	0.303
P23219	PTGS1 COX1	27	0.014	0.301
P31749	AKT1 PKB, RAC	25	0.032	0.328
P00533	EGFR ERBB, ERBB1, HER1	23	0.026	0.296
P37231	PPARG NR1C3	22	0.019	0.327
P05412	JUN	22	0.011	0.309
P25963	NFKBIA IKBA, MAD3, NFKBI	22	0.017	0.293
P09211	GSTP1 FAEES3, GST3	21	0.021	0.326
O15111	CHUK IKKA, TCF16	21	0.019	0.304
O14920	IKBKB IKKB	17	0.007	0.283
P00441	SOD1	17	0.015	0.282
P15692	VEGFA VEGF	16	0.011	0.284

### Prediction of Active Ingredients in LRK

Furthermore, active ingredients from LRK were explored be network pharmacology prediction. As shown in Figure 1B, 24 potential active ingredients directly related to LRK on HMG. According to the OB ≥ 30 and DL ≥ 0.18, 19 active ingredients including saikosaponin c_qt, quercetin, kaempferol, chrysazin, luteolin, beta-sitosterol, isorhamnetin, curcolactone, stigmasterol, 2-methoxy-9,10-dihydrophenanthrene-4,5-diol, areapillin, morin, 3,5,6,7-tetramethoxy-2-(3,4,5-trimethoxyphenyl) chromone, delphinidin, troxerutin, cubebin, α-spinasterol, linoleyl acetate, and vulgaxanthin-I might be responsible for the effect of LRK on HMG, and they were listed in Table 2.

**Table 2:** Information of active ingredients of LRK and their topological parameters.

Compounds	Herbs	PK parameters		Betweenness centrality	Closeness centrality	Degree
		OB (%)	DL			
Saikosaponin c_qt	*Bupleuri Radix*	30.5	0.63	0.1429255	0.3728633	158
Quercetin	*Prunellae Spica*	46.43	0.28	0.1330894	0.3710792	154
Kaempferol	*Bupleuri Radix*, *Prunellae Spica*	41.88	0.24	0.0283762	0.311816	63
Chrysazin	*Polygoni Multiflori Radix*	28.74	0.19	0.0430047	0.3024919	62
Luteolin	*Prunellae Spica*	36.16	0.25	0.0664633	0.3213628	57
Beta-sitosterol	*Cremastrae Pseudobulbus Pleiones Pseudobulbus Cremastrae Pseudobulbus Pleiones Pseudobulbus*, *Curcumae Radix*, *Prunellae Spica*	36.91	0.75	0.0080004	0.2876571	38
Isorhamnetin	*Bupleuri Radix*	49.6	0.31	0.0217248	0.2926011	37
Curcolactone	*Curcumae Radix*	51.51	0.2	0.0473816	0.288728	37
Stigmasterol	*Prunellae Spica*, *Bupleuri Radix*, *Cremastrae Pseudobulbus Pleiones Cremastrae Pseudobulbus Pleiones Pseudobulbus*	43.83	0.76	0.0054813	0.274641	31
2-methoxy-9,10-dihydrophenanthrene-4,5-diol	*Cremastrae Pseudobulbus Pleiones Pseudobulbus*	44.97	0.18	0.0094876	0.2827056	30
Areapillin	*Bupleuri Radix*	48.96	0.41	0.0035804	0.2766548	17
Morin	*Prunellae Spica*	46.23	0.27	0.0068017	0.2666157	17
3,5,6,7-tetramethoxy-2-(3,4,5-trimethoxyphenyl) chromone	*Bupleuri Radix*	31.97	0.59	0.0022551	0.2751281	12
Delphinidin	*Prunellae Spica*	40.63	0.28	0.0016301	0.2708576	8
Troxerutin	*Bupleuri Radix*	31.6	0.28	3.50E-05	0.2329384	7
Cubebin	*Bupleuri Radix*	57.13	0.64	4.26E-05	0.2329773	5
α-spinasterol	*Bupleuri Radix*	42.98	0.76	3.13E-05	0.2601565	4
Linoleyl acetate	*Bupleuri Radix*	42.1	0.2	3.13E-05	0.2601565	4
Vulgaxanthin-I	*Prunellae Spica*	56.14	0.26	1.33E-05	0.2363298	2

### Pathway Analysis of LRK on HMG

The results above showed that 19 active ingredients related to 13 direct targets played the key role of LRK in treating HMG. However, the key pathways still unknown. Therefore, the pathways of the direct target proteins were enriched. The analysis of KEGG pathways indicated that there were five pathways enriched related to HMG, including MAPK signaling pathway, Toll-like receptor signaling pathway, TNF signaling pathway, NOD-like receptor signaling pathway and Chemokine signaling pathway, which were directly involved in inflammation. The proteins like NF-κB (p65), COX2 were involved in major pathways (Table 3). According to the count of relative targets and the percentage on all pathways, MAPK signaling pathway might play the key role in LRK treating HMG. These results indicated that the protect effect of LRK on HMG might associate to MAPK pathway, regulate NF-κB, anti-inflammatory, and anti-oxidative stress.

**Table 3:** Potential pathways and the target proteins in KEGG analysis.

Terms	Count	%	*P*-value	Benjamini	Target protein
MAPK signaling pathway	6	46.2	4.20E-05	2.10E-04	NFKB, JUN, AKT1, IKKA, IKKB, EGFR
TNF signaling pathway	6	46.2	9.70E-09	5.40E-07	NF-κB, AKT1, IKBA, IKKA, IKKB, COX2
Toll-like receptor signaling pathway	6	31.2	5.60E-07	5.70E-06	AKT1, IKKB, IKKA, NF-κB, IKBA, JUN
Chemokine signaling pathway	5	38.5	2.10E-04	7.70E-04	AKT, IKKA, IKKB, NF-κB, IKBA
NOD-like receptor signaling pathway	4	30.8	1.00E-04	4.70E-04	IKKA, IKKB, NF-κB, IKBA

### Therapeutic Effect of LRK on HMG

**FIGURE 2:**
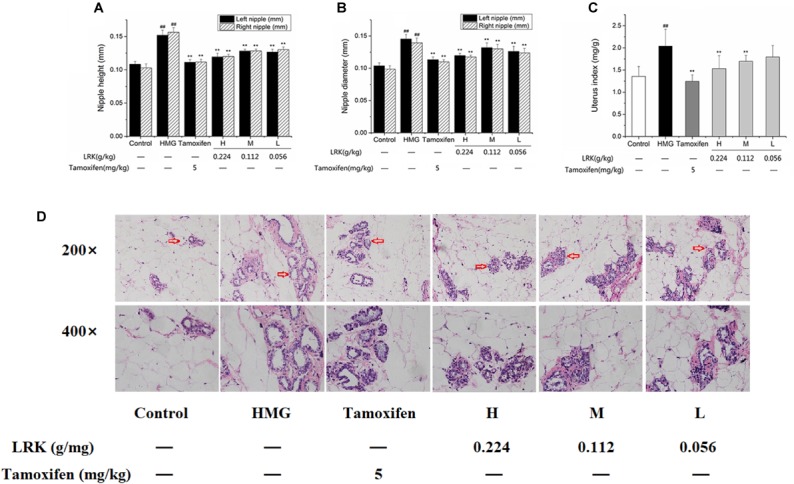
Height and diameter of left and right nipples, uterus index in different group. **(A)** Height of left and right nipples. **(B)** Diameter of left and right nipples. **(C)** Uterus index; *N* = 9; **(D)** The effect of LRK on the pathological features of mammary gland tissues. ^##^*P* < 0.01 vs. Control group; ^∗∗^*P* < 0.01 vs. HMG model group.

In order to assess the therapeutic effect of LRK, the height and diameter of nipples (left 2 and right 2) and uterus index of rats were measured. As shown in Figure 2, both of the left and right nipples were markedly decreased by LRK treatment compared to the HMG model group. Similarly, the diameter of nipples (left 2 and right 2) of rat were also significantly decreased compared to the model group (*P* < 0.01) (Figures 2A,B). And the result showed that the effect of LRK is comparable to the tamoxifen treatment. Uterus is the main target organ of estrogen, the high level of exogenous estrogen will induce the obviously increased of uterus index (Jia et al., 2017). Thereby, the uterus index was examined. It showed that the uterus index was decreased in a dose-dependent manner by LRK treatment (Figure 2C).

Subsequently, hematoxylin and eosin (H&E) staining was performed to visualized the pathological changes of mammary gland sections. As shown in Figure 2D, Estrogen and progesterone stimulation induced severe HMGs in rats, reveled by histological abnormalities, including significant proliferative lesions, mammary ducts ectasia expansion of mammary lumens, significant increase of acinars and lobules. Conversely, the administration of LRK significantly improved the morphological changes of HMGs dose-dependently. Above all, the results indicated the specific therapeutic effect of LRK on HMG.

### Effect of LRK on the Expression of Inflammatory Cytokines (COX-2, IL-1β, iNOS, and TNF-α)

**FIGURE 3:**
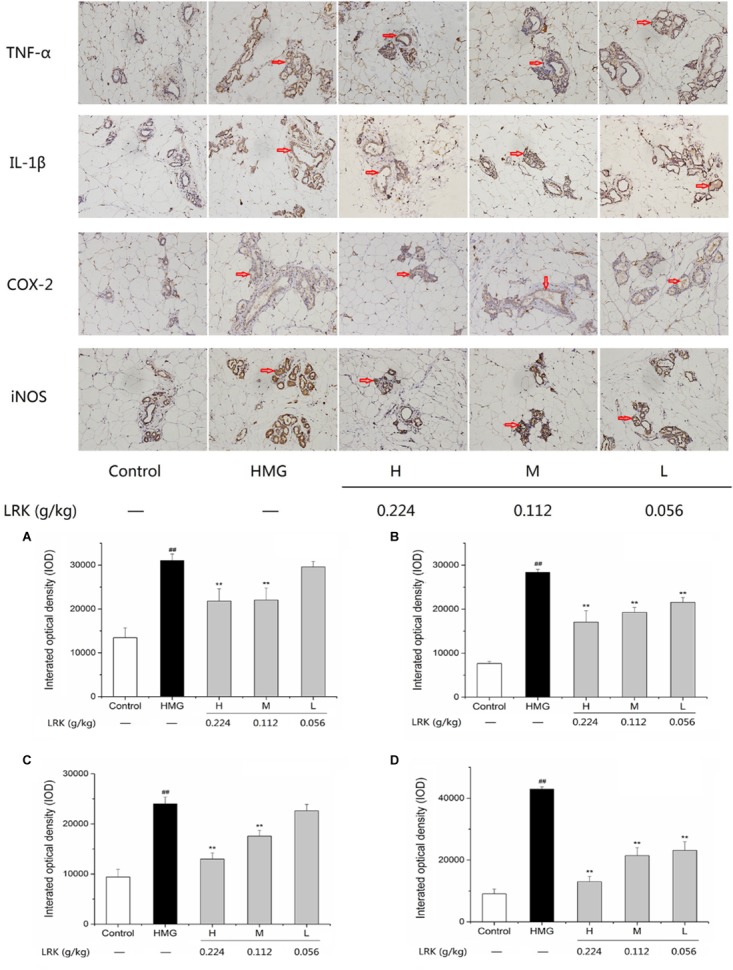
The effect of LRK on the expression of TNF-α, IL-1β, COX-2, and iNOS. The integrated optical density of positive expression of **(A)** TNF-α, **(B)** IL-1β, **(C)** COX-2, **(D)** iNOS; *N* = 3; ^##^*P* < 0.01 vs. Control group; ^∗∗^*P* < 0.01 vs. HMG model group.

Previously studies reported that inflammatory cytokines such as COX-2, IL-1β, iNOS, and TNF-α play important role in the development of HMG (Yang et al., 2013; Chen et al., 2015; Guo et al., 2017). IL-1β and TNF-α, pro-inflammatory cytokines, were significantly increased in model group induced by estrogen and progesterone. While, the LRK treatment could notably decrease the expression of TNF-α and IL-1β compared to model group. COX-2 and iNOS, acted as pro-inflammatory enzymes. LRK treatment significantly inhibited the over-expressions of COX-2 and iNOS in rats induced by estrogen and progesterone in a dose-dependent manner (*P* < 0.01) (Figure 3). In summary, the results showed the significant anti-inflammatory effect of LRK.

### Effect of LRK on Oxidative Stress [8-OHdG and Nitrotyrosine (NT)]

**FIGURE 4:**
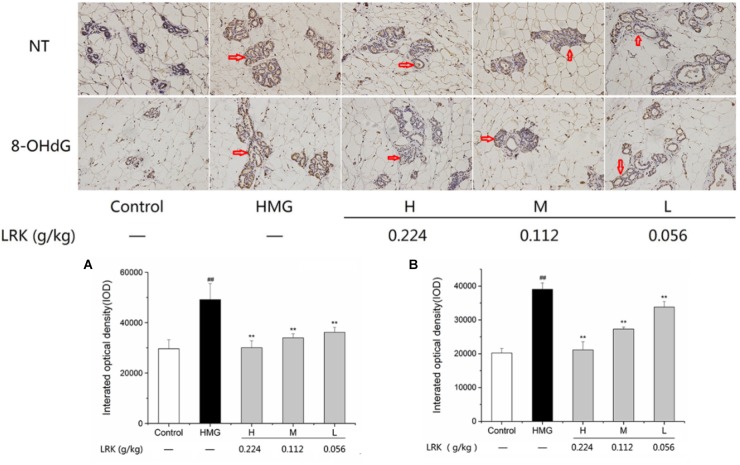
The effect of LRK on the expression of NT and 8-OHdG. The integrated optical density of positive expression of **(A)** NT, **(B)** 8-OHdG in different group; *N* = 3; ^##^*P* < 0.01 vs. Control group; ^∗∗^*P* < 0.01 vs. HMG model group.

As shown in Figure 4, there was an obvious oxidative stress injury in model group, reveled by the high expression of 8-OHdG and NT, markers of oxidative stress, compared to the control group. In the LRK groups, the high expression of 8-OHdG and NT were significantly inhibited in a dose-dependent elevation, which indicated the anti-oxidative stress effect of LRK (Figure 4).

### Effect of LRK on Expression of ERα and PR

**FIGURE 5:**
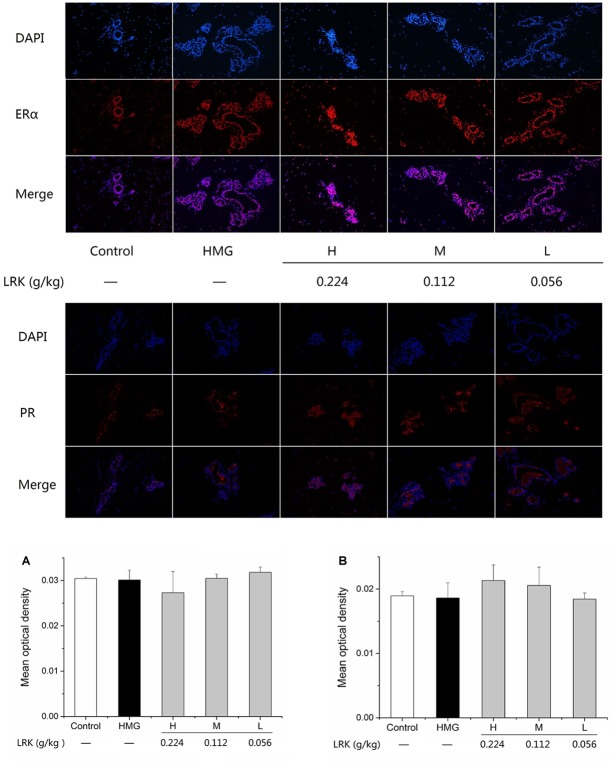
The effect of LRK on the expression of ERα and PR. The mean optical density of positive expression of **(A)** ERα, **(B)** PR in different groups; *N* = 3.

The expression of estrogen receptor and progesterone receptor in mammary gland plays an important role in the physiological and pathological changes of mammary gland. So, we examined the expression of ERα and PR. As shown in Figure 5, LRK could not affect the expression of ERα and PR.

### Effect of LRK on Expression of p-JNK, p-P38, p-ERK, and NF-κB (p65)

**FIGURE 6:**
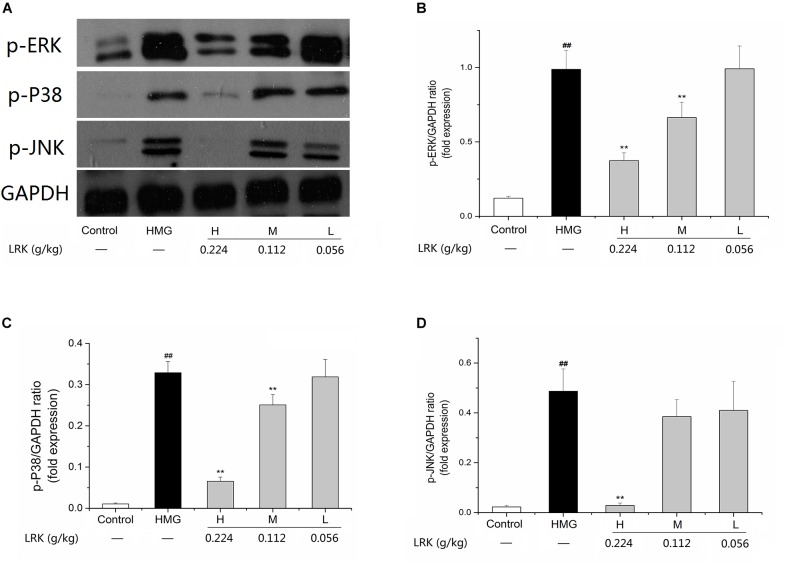
Alterations of the mitogen-activated protein kinase (MAPK) pathways in the mammary glands of LRK-treated rats. **(A)** Expression of p-ERK, p-P38, and p-JNK by western blot analysis; **(B)** The p-ERK/GAPDH ratio; **(C)** the p-P38/GAPDH ratio; **(D)** the p-JNK /GAPDH ratio. Sample loading was normalized by GAPDH. *N* = 3; ^##^*P* < 0.01 vs. Control group; ^∗∗^*P* < 0.01 vs. HMG model group.

The pathway analysis results in this study indicated that LRK may play anti-inflammatory and anti-oxidative stress role through MAPK/ NF-κB signaling pathways. To determine whether LRK could affect MAPK, we examined the expressions of p-JNK, p-P38, p-ERK. The results showed that p-P38, p-ERK, and p-JNK were slightly expressed in normal mammary cells. In HMG model group, expressions of p-P38, p-ERK, and p-JNK were notably increased. LRK administration significantly suppressed the over-expressions of p-P38, p-ERK, and p-JNK versus the model group (Figure 6).

**FIGURE 7:**
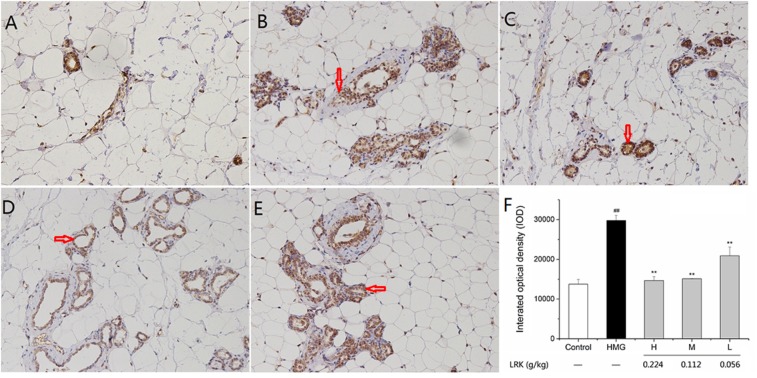
The effect of LRK on the expression of NF-κB (p65). Immunohistochemical analysis of NF-κB (p65) in **(A)** Control group, **(B)** HMG Model group, **(C)** H group, **(D)** M group, **(E)** L group, **(F)** The integrated optical density of positive expression of P65; *N* = 3; ^##^*P* < 0.01 vs. Control group; ^∗∗^*P* < 0.01 vs. HMG model group.

There existed a large degree of cross-talk within the MAPK cascades and other signaling networks. For example, there were interactions between mediators of the MAPK and NF-κB pathway (Chen et al., 2015). The expression of NF-κB (p65) was examined by immunohistochemical analysis. NF-κB (p65) was increased significantly in the HMG model group. LRK treatment significantly and dose-dependently suppressed the mammary over-expressions of NF-κB (p65) when compared to the model ones (Figure 7).

## Discussion

HMG, a common disease in middle-aged women has severe cancerous tendencies, and poses a significant public health challenge to women (Marchant, 2002). It is reported that HMG, especially atypical HMG, leads to higher risk of breast cancer. (Wang et al., 2011; Zhang et al., 2012). Studies showed that HMG is related to endocrine disorders, the most of that are caused by the imbalance of estrogen and progestin. (Liu et al., 2012; You et al., 2017) The treatment of HMG has become a heated topic in the world. HMG is mainly treated by surgery and medication (Henry, 2014; Chen et al., 2015). Tamoxifen, as the main therapeutic drug of HMG could improve the overall survival for patients. However, adherence to and persistence with the medications is poor in part because of bothersome side effects that can negatively affect quality of life (Henry, 2014). Thus, it is important and urgency to find more effective and few side effects drug to block development of HMG.

LRK, a TCM formula, has been clinically used for the treatment of HMG for several years. The First Affiliated Hospital of the General Hospital of PLA conducted clinical trials and demonstrated that LRK could inhibit HMG (Li et al., 2013). Previous study showed high effective rate (88.0%) in the treatment of HMG. Meanwhile the patients’ symptoms and abnormalities of gonadal hormone was obvious improved (Qian et al., 2007). However, the underlying mechanisms of LRK for HMG are still unclear. Thus, the aim of this study is to explore the underlying mechanism of LRK on HMG.

To clarify the effect of LRK on HMG in rats, we established HMG model in rats induced by estrogen and progesterone. The decreasing of nipple heights and diameters, uterus index and ameliorate of histopathological in LRK groups indicated that LRK could improving HMG in rats. Then, we further explore the active ingredients and underlying mechanism of LRK on HMG.

To reveal the therapeutic mechanisms of LRK on HMG, we firstly predicted the active ingredients and potential targets of LRK against HMG through network pharmacology. We found 19 potential compounds, including saikosaponin c_qt, quercetin, kaempferol and chrysazin, Luteolin, beta-sitosterol, isorhamnetin, curcolactone, stigmasterol, 2-methoxy-9,10-dihydrophenanthrene-4,5-diol, areapillin, morin, 3,5,6,7-tetramethoxy-2-(3,4,5-trimethoxyphenyl) chromone, delphinidin, troxerutin, cubebin, α-spinasterol, linoleyl acetate and vulgaxanthin-I might play the key role in LRK-treated HMG. Studies showed that most of those potential active ingredients have anti-inflammatory or anti-oxidative effects, such as quercetin, (Song et al., 2018, kaempferol (Huang et al., 2018; Wang J. et al., 2018), Luteolin (Aziz et al., 2018), beta-sitosterol (Liao et al., 2018), isorhamnetin (Qiu et al., 2016), delphinidin (Wang et al., 2017), troxerutin (Najafi et al., 2018), cubebin (Bastos et al., 2001), morin (Athira et al., 2016), linoleyl acetate (Peana et al., 2002) have anti-inflammatory effects. Compounds like quercetin (Sherif, 2018), kaempferol (Kouhestani et al., 2018), Luteolin (Yang et al., 2018), beta-sitosterol (Yin et al., 2018), delphinidin (Cheol et al., 2016), troxerutin (Farajdokht et al., 2017), morin (Ma et al., 2016), linoleyl acetate (Peng et al., 2014) showed anti-oxidative stress effect. Studies indicated that HMG was related to inflammatory and oxidative stress (Chen et al., 2015). Therefore, those active ingredients predicted by network pharmacology might play important role in LRK treat HMG.

Meanwhile, KEGG analysis indicated that MAPK signaling pathway contributes the most for the therapeutic effect of LRK on HMG, it might be the essential pathway for our research. Additionally, Toll-like receptor signaling pathway and TNF signaling pathway were either directly involved in inflammation or played other important roles.

Simultaneously, there were studies proved that MAPK signaling pathway is essential in regulating many cellular processes including inflammation, cell stress response, cell differentiation, cell proliferation, metabolism, motility and apoptosis (Arthur and Ley, 2013). There were four distinct MAPK cascades including the extracellular signal-regulated kinase (p42/44 ERK)/MAPK, the p38 pathway, the c-jun N-terminal kinase (JNK)/stress-activated protein kinase (SAPK) pathway and the Big MAP kinase-1 (BMK-1) pathway (Burotto et al., 2014). P38, ERK, and JNK pathway was the most important signaling pathways involving the regulation of many physiological functions of cells and playing an important role in the anthogenesis and pathophysiological process of many kinds of diseases, such as regulating the proliferations and differentiations of cells, participation in oxidative stress reaction (Li et al., 2017). Currently, more and more researches proved that TCM have the protective effect against HMG via the possible mechanism of regulating endocrine, inflammatory and oxidative stress (Wang et al., 2011; Zhang et al., 2012). And studies indicated that TCM can significantly inhibit and improve HMG in a large degree via ERK, JNK signaling pathway, as well as regulating NF-κB (Squires et al., 2003; Chen et al., 2013; Wu, 2015). It is well-known that NF-κB can activate the inflammatory cytokines like IL-1, TNF-α, and COX2 (May and Ghosh, 1998). Researches indicated that MAPK and NF-κB signaling pathway play an important role in HMG treated by TCM while NF-κB building a link among oxidative stress and inflammatory responses in HMG (Chen et al., 2015). Therefore, we verified the MAPK and NF-κB signaling pathway for further experiments.

The experimental results showed that the phosphorylation of P38, ERK, and JNK in HMG rats was significantly increased after stimulation of estrogen and progesterone. While the overexpression of p-p38, p-ERK, and p-JNK were decreased after the treatment of LRK. As the downstream of MAPK signaling pathway and an important inflammatory transcription factor, NF-κB was decreased after LRK treated. And the expression of inflammatory mediators (including IL-1, and TNF-α) and pro-inflammatory cytokines (such as COX2 and iNOS) were down-regulated after LRK treated. Those results indicated LRK could reduce inflammation in HMG.

ER and PR, as the receptor of estrogen and progestin, and studies showed PR and ERα could active the MAPK signaling pathway (Liu, 2012). The present study showed that LRK treatment has no effect on the expression of ERα and PR. Those results indicate that LRK might plays therapeutic effect on HMG by activating MAPK signaling pathways directly, rather than through ERα and PR to activating MAPK signaling pathways.

8-OHdG is a predominant form of free radical-induced oxidative lesions in nuclear and mitochondrial DNA. Therefore, 8-OHdG is widely used as a biomarker for oxidative stress (Wu et al., 2004; Arulselvan et al., 2016). Nitrotyrosine (NT) is mainly formed by the reaction of superoxide radicals with NO, which is used to detect peroxynitrite (Fitri et al., 2017). NT formation has been detected in a variety of systemic inflammatory diseases and is considered as a marker of NO-derived species. Estrogen can generate considerable reactive oxygen species (ROS) and lead to high level of oxidative stress (Chen et al., 2015; Das Gupta et al., 2015). Activation of NF-κB could upregulated the expression of NO, further promote ROS and oxidative stress indexes (Qi, 2014). In the present study, LRK treatment significantly reduced the expression of 8-OHdG and NT. Hence, LRK may also act as an anti-oxidative agent on HMG treatment.

Our data indicated that LRK treatment protects the mammary glands from the damage of oxidative stress and inflammation via suppresses of MAPK/NF-κB signaling pathways without affecting on the expression of ERα and PR.

In this study, there were some limitations that several pathways were predicted related to the therapeutic effect of LRK on HMG via network pharmacology, however, parts of pathway were demonstrated by our experiment. Although, 19 potential active compounds were predicted by network pharmacology, and literatures reported showed anti-inflammatory and anti-oxidative effect of those ingredients, the effect of those active ingredients on HMG were still unknown. Maker clear the active ingredients is necessary for the formula research. Accordingly, in the next work of our group, we will have a comprehensive study to illuminate the active ingredients and the mechanisms of LRK against HMG.

Previous study verified the effect of MAPK inhibitor (UO126) in mammary epithelial cells (HC11 and EpH4) and breast cancer cells (MC4-L2), indicated that MAPK inhibitor could inhibit the activation of ERK signaling and suppress the proliferation, to inhibiting hyperplasia and growth (Cotrim et al., 2013). Our research group will further validate the effect of MAPK/NF-κB signaling pathway in LRK treat HMG by using the MAPK inhibitors to mimic the function of LRK, to proving the importance of MAPK/NF-κB signaling pathway for LRK work in HMG rat. Furthermore, we will comprehensively explicit the active ingredients of LRK by screening the active ingredients of LRK *in vitro* alternative model, and testing the active components to see whether they could achieve the similar effects as LRK treatment subsequently.

## Conclusion

**FIGURE 8:**
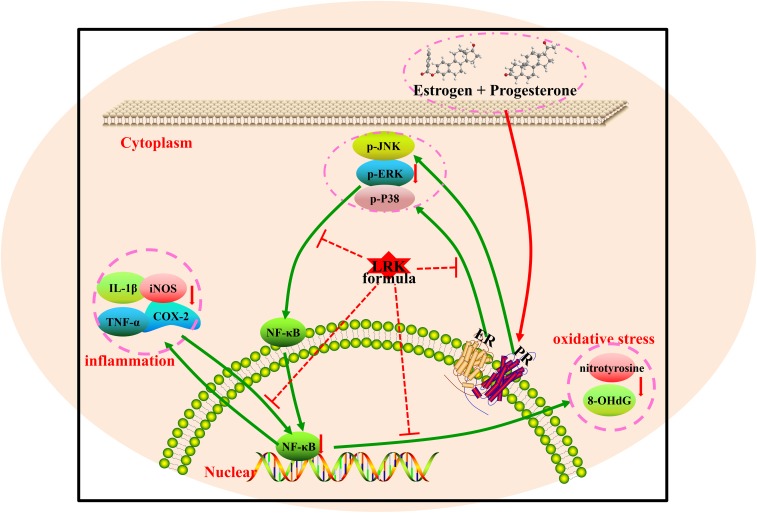
The probable protection mechanism of LRK in HMG.

Summarily, the present study demonstrated that there was a therapeutic effect of LRK on HMG by reducing pathological lesions in mammary gland tissue attenuating the over-activation of inflammation cytokines such as IL-1β, TNF-α, COX-2, iNOS, and suppressing the expression of 8-OHdG and NT. Furthermore, the combination of network pharmacology and experiment verification illustrated that the protect effect of LRK on HMG in rats might be associated with MAPK/NF-κB signaling pathways (Figure 8).

## Author Contributions

YW was responsible for primary data generation, analysis and writing the manuscript. SW, XZ, LQ, YZ, and WZ participated in the design of the study. YY, XL, and TW were involved the *in vivo* experimentation and technical work. SW and YY were responsible for the extensive statistical analyses. HL, TG, WZ, and XZ gave advice on the writing.

## Conflict of Interest Statement

The authors declare that the research was conducted in the absence of any commercial or financial relationships that could be construed as a potential conflict of interest. The handling Editor and reviewer YH declared their involvement as co-editors in the Research Topic, and confirm the absence of any other collaboration.

## Footnotes

1 http://tcm.cmu.edu.tw/

2 http://lsp.nwu.edu.cn/

3 http://lifecenter.sgst.cn/hit/

4 http://bidd.nus.edu.sg/group/cjttd/

5 https://www.omim.org/

6 http://dip.doe-mbi.ucla.edu/dip/Main.cgi
